# AI-assisted reliability assessment for gravure offset printing system

**DOI:** 10.1038/s41598-022-07048-z

**Published:** 2022-02-22

**Authors:** Anton Nailevich Gafurov, Thanh Huy Phung, Inyoung Kim, Taik-Min Lee

**Affiliations:** 1 Department of Printed Electronics, Korea Institute of Machinery and Materials, 156, Gajeongbuk-ro, Yuseong-gu, Daejeon, 34103 Republic of Korea; 2 Department of Nanomechatronics, University of Science and Technology, 217, Gajeong-ro, Yuseong-gu, Daejeon, 34113 Republic of Korea

**Keywords:** Mechanical engineering, Techniques and instrumentation

## Abstract

In printed electronics, flawless printing quality is crucial for electronic device fabrication. While printing defects may reduce the performance or even cause a failure in the electronic device, there is a challenge in quality evaluation using conventional computer vision tools for printing defect recognition. This study proposed the computer vision approach based on artificial intelligence (AI) and deep convolutional neural networks. First, the data set with printed line images was collected and labeled. Second, the overall printing quality classification model was trained and evaluated using the Grad-CAM visualization technique. Third and last, the pretrained object detection model YOLOv3 was fine-tuned for local printing defect detection. Before fine-tuning, ground truth bounding boxes were analyzed, and anchor box sizes were chosen using the k-means clustering algorithm. The overall printing quality and local defect detection AI models were integrated with the roll-based gravure offset system. This AI approach is also expected to complement more accurate printing reliability analysis firmly.

## Introduction

Flexible or hybrid electronics^1^ are the current trends in the electronics production industry. They reduce cost by saving raw materials and increasing output while using thinner polymer substrates and adapting roll-to-roll techniques. The roll-to-roll technique is especially advantageous when combined with printing processes^2^, which allow for the selective application of functional materials and the possibility to pipeline the whole fabrication process into a single workflow for multilayer device fabrication, further reducing the cost.

The gravure offset is one of the printing techniques used for manufacturing various electronics, such as silver grid transparent electrodes^3^, pressure sensors^4^, and planar inductors^5^, mainly through fine line patterning, because of the following reasons. First, the plate-making process allows for fabricating the gravure printing roll or printing plate with much finer resolution down to several micrometers^6,7^, unlike other conventional printing techniques, such as widespread screen printing, which has at least one order of magnitude lower resolution. Second, as gravure offset printing evolved from the so-called pad printing suitable for printing electronics onto nonplanar surfaces, such as electroluminescent displays (ELDs)^8,9^ and radio-frequency identification (RFID) antennas^10^, it inherited its main feature: the pad-like blanket made of silicone polymer and wrapped around a cylinder. This blanket cylinder allows for printing on rigid substrates and improves ink transfer and printing quality.

**Figure 1:**
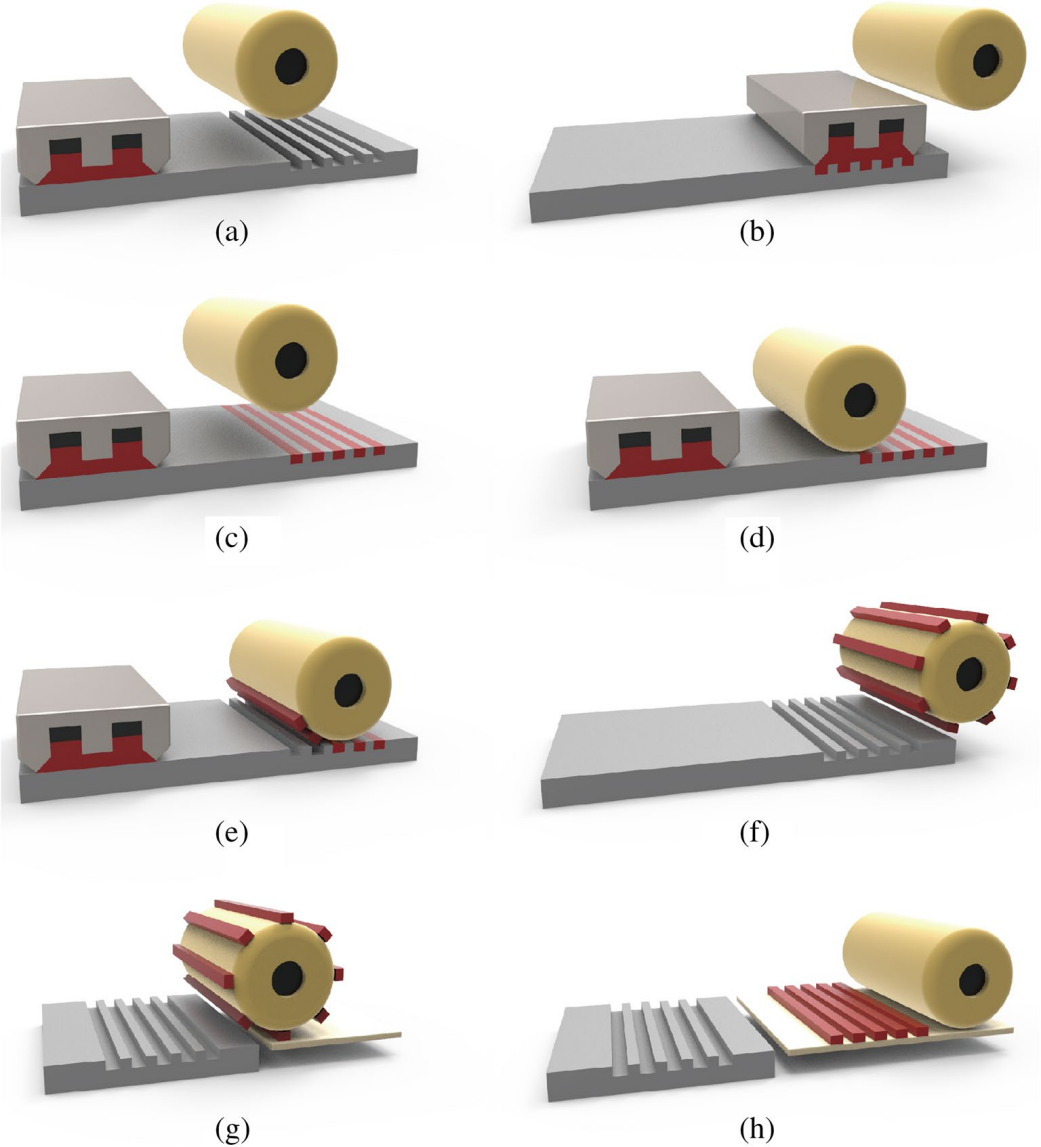
The gravure offset printing process: (**a**, **b**) ink filling the gravure plate and (**c**) ink doctoring, (**d**, **e**, **f**) off step when ink is transferred from the gravure plate to the blanket roll, (**g**, **h**) set step when ink is transferred from the blanket roll to the substrate.

**Figure 2:**
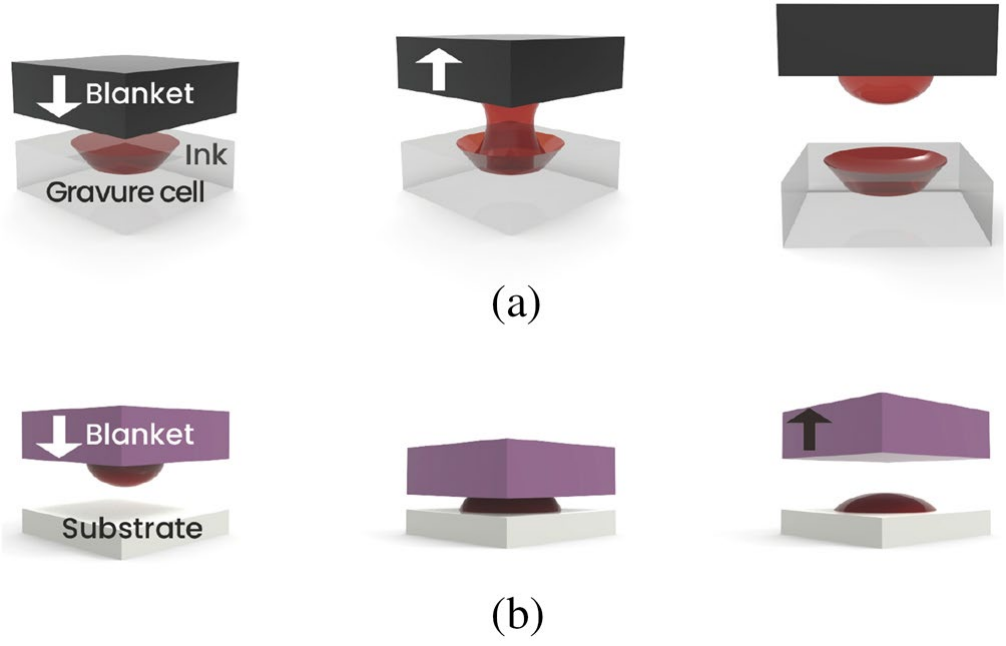
Ink transfer mechanism in gravure offset printing: (**a**) off step, (**b**) set step.

The gravure offset printing process consists of three steps: ink filling and doctoring, as well as the off and set processes, as shown in Fig. 1. First, the ink is filled into the recessed printing elements of a gravure printing cylinder or a printing plate and then doctored with a sharp doctor blade made of ceramic or stainless steel. Second, the ink is transferred from the gravure cylinder or plate to the offset blanket, which is the off process. Third and last, the ink is transferred from the blanket to the substrate, called the set process. This transferring mechanism (Fig. 2) is carried out through applied pressure and is a key factor of printing quality, which may cause the following defects to form: printed line width gain, bulge outs, and bad surface roughness. These defects occur for several reasons. The ink is most likely split in half during both the off and set steps, leaving the blanket contaminated by excess ink after the set step. Because the printing process is continuously repeated, the residual ink on the blanket can interfere with new printing and form a printing defect. Huang et al.^11^ modeled the amount of ink left on the blanket from the previous print based on the balance between the cohesive and adhesive forces of the ink concerning the blanket, substrate, and gravure plate. When the contact angle of the blanket is increased or reduced of its free surface energy, the ink residuals also become minimal. Kang et al.^12^ proved these simulations and investigated the experimental methods of adjusting the blanket and substrate ink wettability through various physical and chemical approaches. The most crucial step is to achieve 100% transfer during the set stage, which can be ensured when both the cohesive force within the ink and its adhesive strength to the substrate are higher than its adhesion to the blanket.

**Figure 3:**
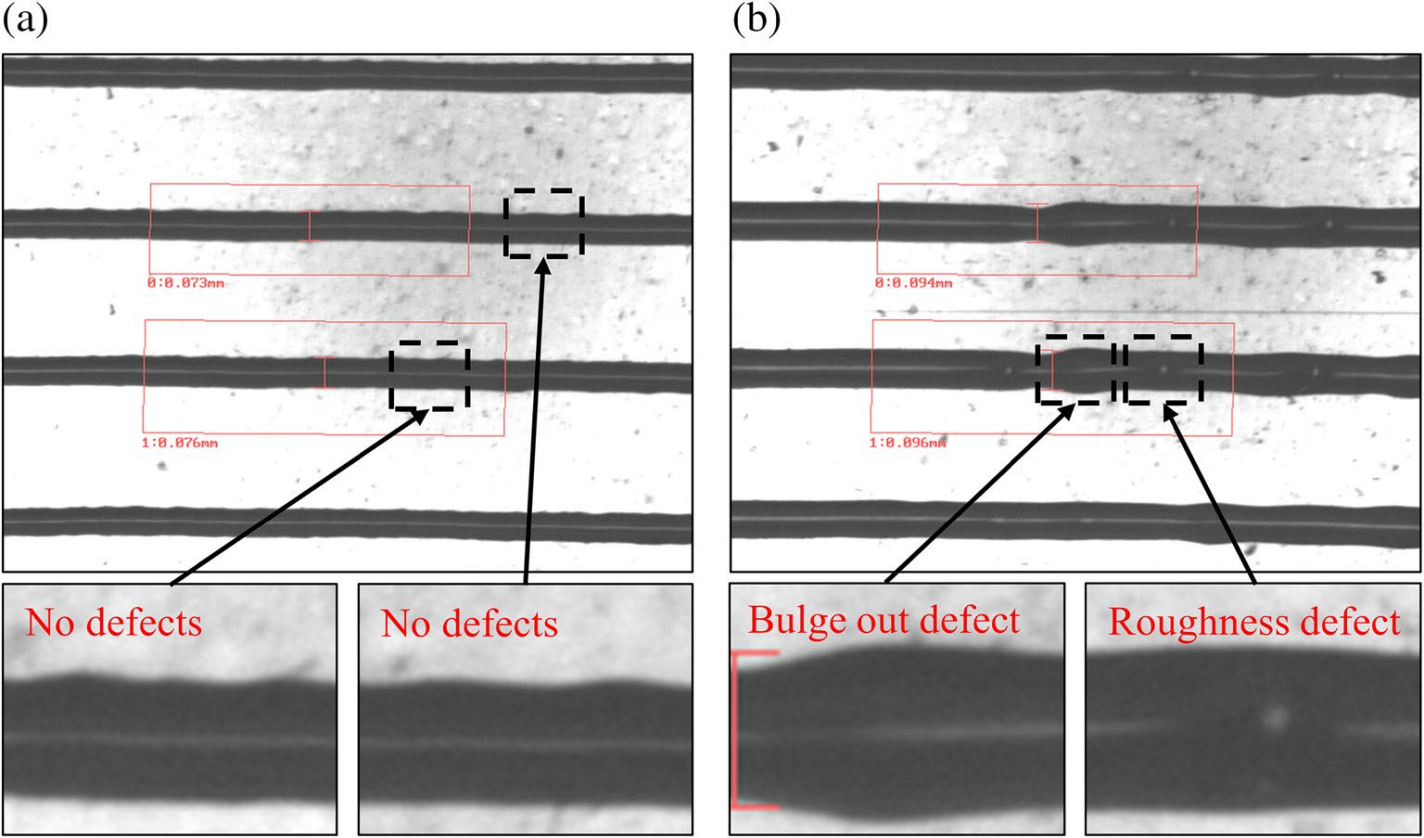
Images of printed lines captured by the camera: (**a**) printed lines without defects, (**b**) with defects.

Despite the use of polydimethylsiloxane (PDMS) with low free energy as a blanket, as well as the activation of the substrate to promote adhesion, the major sources of defects are the properties of the printing ink. The ink’s main property is viscosity, which directly affects the cohesive forces inside the printing layer and, thus, should be controlled during printing. In gravure offset printing, the ink should easily fill in and be released from the engraved cells, achievable with very low viscous inks but compromised by the weakening of its cohesive forces. Shen et al.^13^ investigated the temperature effect and solvent content of the ink that, as known, affect viscosity. Pudas et al.^14^ also proposed the composition of ink with higher viscosity and achieved 100% ink transfer from the blanket while it could still be released from the gravure cells. In addition, Lee et al.^15^ studied gravure offset printing under various printing conditions that revealed the primary aspect of the gravure offset printing reliability—printed line width to the ink solvent absorption by the PDMS blanket. First, this absorption amount depends on how long the PDMS blanket is in contact with the ink during ink transferring, thus determining the contact angle between the two. This change in contact angle, in turn, determines the width of the printed lines. Second, while ink transferring, the blanket partially absorbs the ink solvent, increasing viscosity. However, during the print run, the PDMS blanket becomes saturated by the ink solvent, which causes its absorbing ability to decrease, and the viscosity of the following ink portion is not being tailored anymore. This leads the ink to be split in half during the set process, and its residuals are left on the blanket, causing bulges and roughness with local defects in the subsequent prints (Fig. 3). Kim et al.^16^ adopted the PDMS blanket swelling control technique with an air blowing unit with humidity- and temperature-controlled airflow, thereby facilitating the evaporation of the solvent from the PDMS blanket. These actions enhanced the gravure offset printing reliability, which was evaluated through the line width measured using a digital camera. However, if the line width is within tolerance during consecutive printing but other defects are present, the failure regime of the printing should be detected, which becomes tricky when using the conventional tools^17,18^ of computer vision.

The modern computer vision approach involves artificial intelligence (AI) that includes deep neural networks (DNNs). With a combination of convolutional layers^19^ and skip connections^20^, it became possible to train extreme DNNs that can resolve highly nonlinear tasks, such as image classification or object detection. These approaches may help with reliability investigation by estimating the printed pattern either qualitatively by classifying whether the whole image satisfies the excellent quality criteria or quantitatively by detecting the number of local printing defects categorized by class. Ultimately, the qualitative approach aims to define the reliability failure in a binary manner, and as a stand-alone system, it might not be informative enough. When it is complemented by local defect detection, the reliability can be expressed numerically.

Various AI approaches were used for quality inspection for electronics production in the fields of printed circuit boards (PCBs)^21,22^ and solar cell fabrication^23,24^. Adibhatla et al.^21^ adopted a transfer learning technique to train YOLOv2 object detection for PCB defect recognition. Wei et al. ^22^ developed the convolutional neural network (CNN) model to compare a manufactured PCB with a reference. Meanwhile, Chen et al. ^23^ proposed a multispectral CNN approach for solar cell surface inspection, and Zhang et al.^24^ developed a surface defect detector.

The present study aims to develop a printing quality evaluation system for printing reliability based on contemporary AI computer vision techniques. The data set composed of images with conductive lines printed using gravure offset was created. A Visual Geometry Group (VGG)–like classification DNN model with skip connections was trained to classify the overall printing quality. Then, a pretrained YOLOv3 object detection model was fine-tuned using transfer learning to detect local printing defects.

### Experimental setup

The roll-based gravure offset machine was used, as described in the previously published paper^25^. It was designed as an on-table device and included the following parts: the unwinder, offset roller with two load cells, cup-type doctoring blade, gravure plate, stage, furnace, rewinder, and charge-coupled device (CCD) camera. During printing, the stage with the gravure plate, unwinder, rewinder, and furnace move laterally, while the offset roller is kept stationary and rotates synchronously with the stage movement. The printing pressure is controlled by positioning the offset roller vertically and measuring the pressure by two load cells. After each printing, the images of the printed pattern are captured by the camera with a resolution of 2.41 µm/pixel while fixed by vacuum to the stage surface. Then, these images are processed using conventional machine vision techniques for line width measuring and saved into the report file. The printing ink is composed of spherical silver particles with 0.3–1.0 µm, and the composition consists of 85.5% silver particles, 7.3% polymer, 6.7% solvent, and 0.5% inorganic adhesion promoter. In addition, the PDMS offset blanket XR-3003 (Dow Corning Korea Ltd.) was used. The printing conditions are summarized in Table 1.

**Table 1:** Gravure offset printing conditions.

Ink	Silver nanoparticle ink with 0.3–1.0 µm particle size
Blanket	XR-3003, Dow Corning Korea Ltd
Printing pressure	5.5–6.0 kgf
Printing speed	50 mm/s

**Figure 4:**
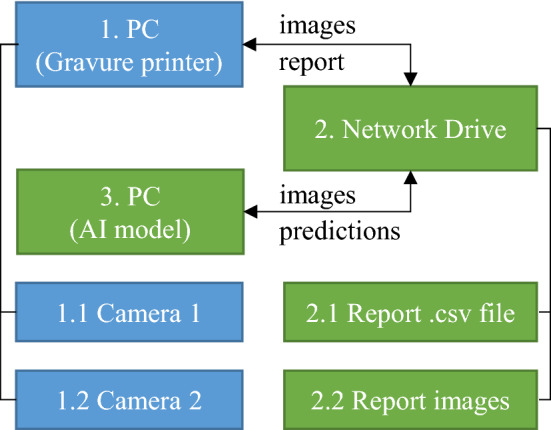
The scheme of AI model integration with the gravure offset system.

Developed AI models described in this paper has been combined with this gravure-offset printing machine for predictions. Both overall printing quality classification and local printing defect detection AI models were integrated through Ethernet connection. The scheme of the AI model integration is shown in Fig. 4. First, the gravure offset PC (part 1) acquires the images from cameras (parts 1.1 and 1.2) and saves them to the designated folder onto the network drive (part 2). Second, the PC with a running AI script (part 3) reads the images, sends them through the AI models for processing, and saves the generated predictions as .csv files (part 2.1) and report images (part 2.2) onto the network drive hosted by the PC with an AI script. Third and last, the gravure offset PC reads the .csv report files stored in the network drive and compiles them into the final report, also stored in the network drive.

### Data set

During printing, images of the printed lines were captured. These images were evaluated and labeled based on their overall quality and the presence of local defects (Fig. 3). Aside from line width gain, the following defects were present in the captured images: bulge outs and nonuniform line roughness, which, as discussed earlier, originated from blanket contamination and were inherent to the liquid ink transferring. There are 299 images in total, which were divided into 2 classes: 225 with satisfactory quality and 74 with defects (Table 2). For local defect detection, all 74 images with distinctive local defects were chosen and labeled using the LabelImg tool for object detection. For validation purposes, 25% of the data set for classification and 20% for object detection performance were designated.

**Table 2:** Image data set distribution for both image classification and object detection tasks.

Category	No. of images for image classification			No. of images for object detection
	Total	With defects	Without defects	With defects
Training data set	209	50	159	59
Validation data set	90	24	66	15
Total		74	225	74

### AI model for overall printing quality classification

**Figure 5:**
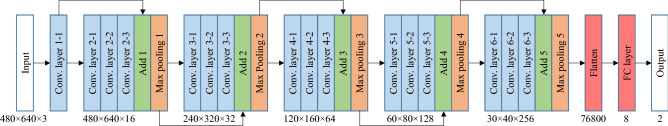
DNN model for image classification: VGG-16 like structure with skip connections.

For the image classification task, the DNN model with skip connections, as shown in Fig. 5, was built and trained from scratch using the data set, described in Sect. 3. Augmentation was performed on each batch, including random brightness, contrast, zoom, rotation augmentation layers, and random horizontal and vertical flip, to enlarge the data set. Since the data set is imbalanced considering number of images per class, class weights were applied to categorical loss. The model parameters are summarized in Table 3, and the programming was done using TensorFlow and Keras frameworks in Python.

**Table 3:** Summary of the image classification model parameters.

Kernel initializer		He normal
Activation		CNN layers and dense layer – ReLU Output layer – Softmax
Optimizer		Adam (learning rate = 0.0005)
Batch size		32
Loss function		Categorical cross-entropy with class weights
Data augmentation	Image brightness	+ 40 – + 70
	Image contrast	1.0–1.5
	Image flip	Horizontal and vertical
	Zoom in	0.0–0.2
	Rotation	± 90° Filling mode: constant (255)

**Figure 6:**
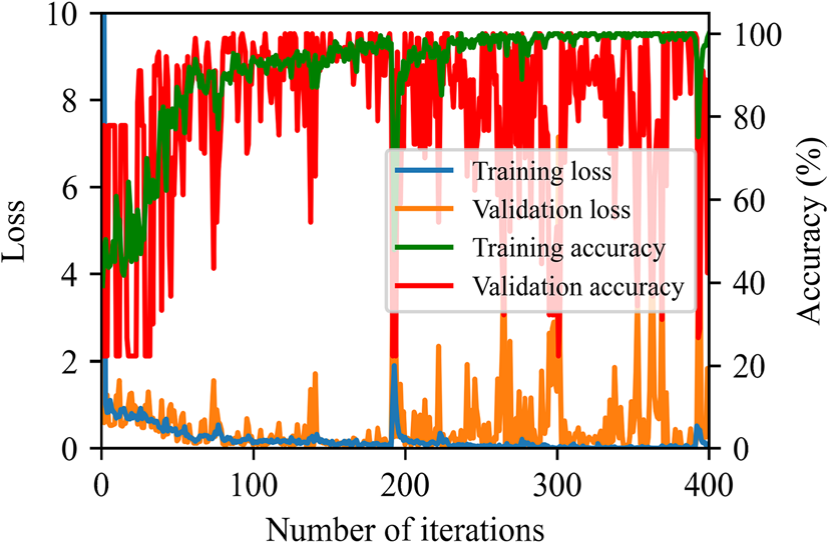
The training process of the image classification model: training and validation accuracy, and training and validation loss.

The training process is shown in Fig. 6. It was done for 400 epochs, and the model weights, which showed the best validation accuracy results, were used for predictions.

**Figure 7:**
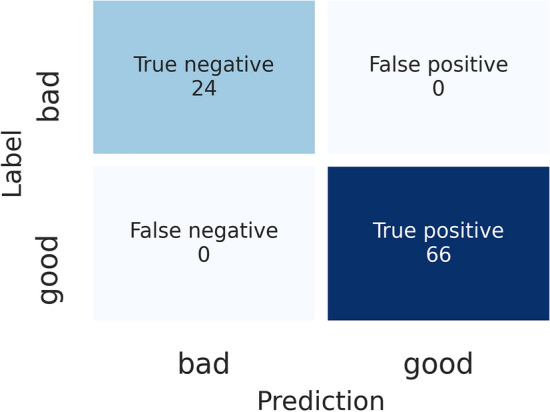
Confusion matrix for overall printing quality validation data set.

**Figure 8:**
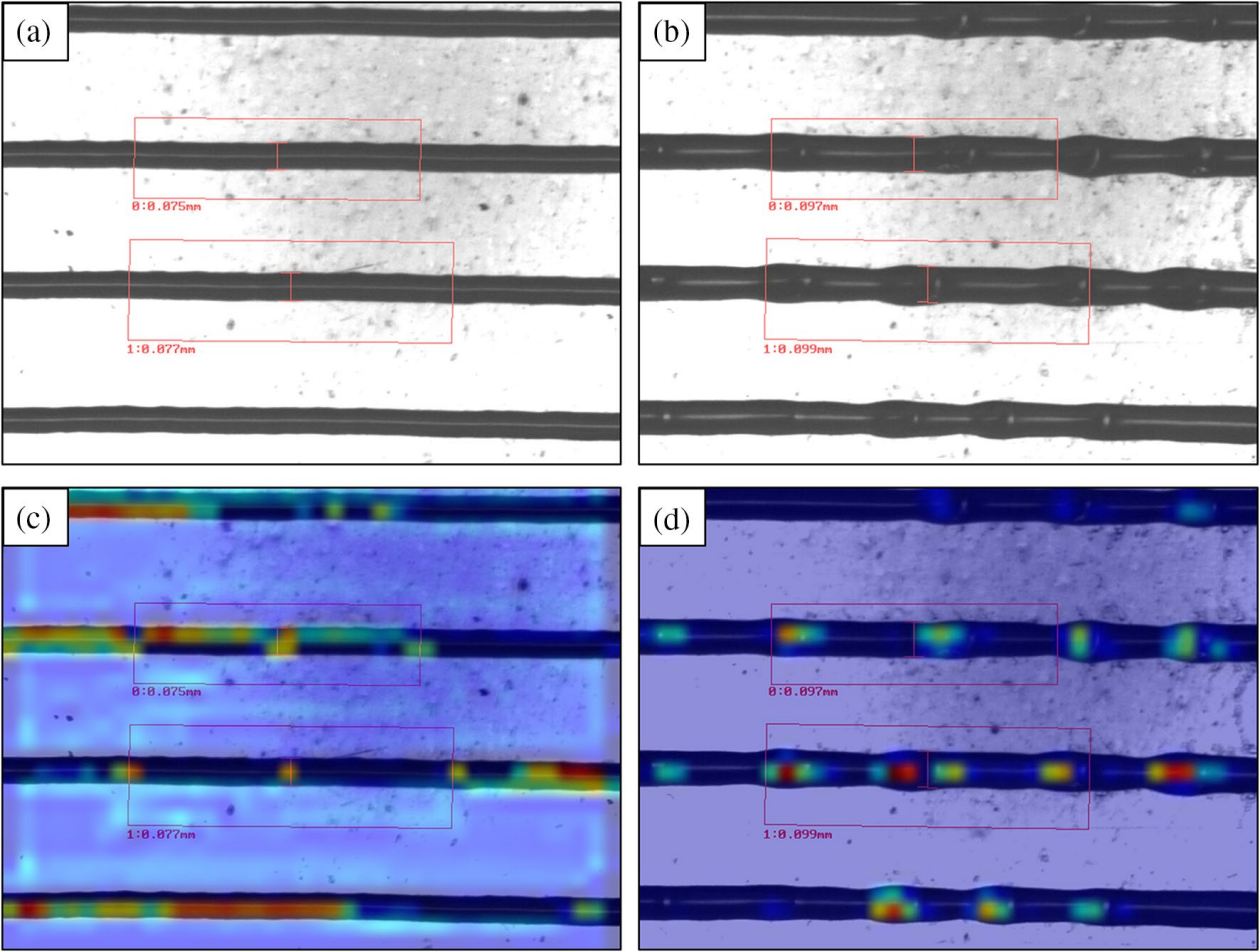
Grad-CAM images. The original images of printed lines (**a**) without and (**b**) with defects and superimposed with the Grad-CAM heat map’s original images for (**c**) printed lines without defects and (d) printed lines with defects.

The model was evaluated using the validation data set, and the results were represented as a confusion matrix, as shown in Fig. 7. In reinsuring the model performance, the Grad-CAM^26^ approach was utilized to visualize the trained model performance. In this algorithm, using the last convolutional layer inputs (“add 5” layer) concerning model predictions, it is possible to know which parts of the image triggered this decision. Then, the results were represented in a heat map of 30 × 40 pixels (width × height) and superimposed with the original image (Fig. 8a,b). Figure 8c,d shows the overall printing quality model’s attention regions involved in the corresponding class assignment. It shows that the model pays attention to the lines rather than to the substrate, and for the defective images, the actual defects areas are of the highest interest to the model when assigning the corresponding class.

### AI model for local printing defects detection

The fine-tuning approach was applied to retrain the YOLOv3 mode^27^ with pretrained weights. This model is designed to predict objects’ position by proposing bounding boxes position in 416 × 416 pixel images. This model has several features. First, unlike models based on region-proposed algorithms, the predictions are made in one step, increasing detection speed. Second, the predictions are computed based on three feature maps from different layers, which increases model accuracy. Third and last, the training process includes predefining anchor boxes, which indicate to the model the expected object size in the analyzed image. After obtaining the best epoch weights, the model was used to predict defects present in the newly captured images.

### K-means clustering

**Figure 9:**
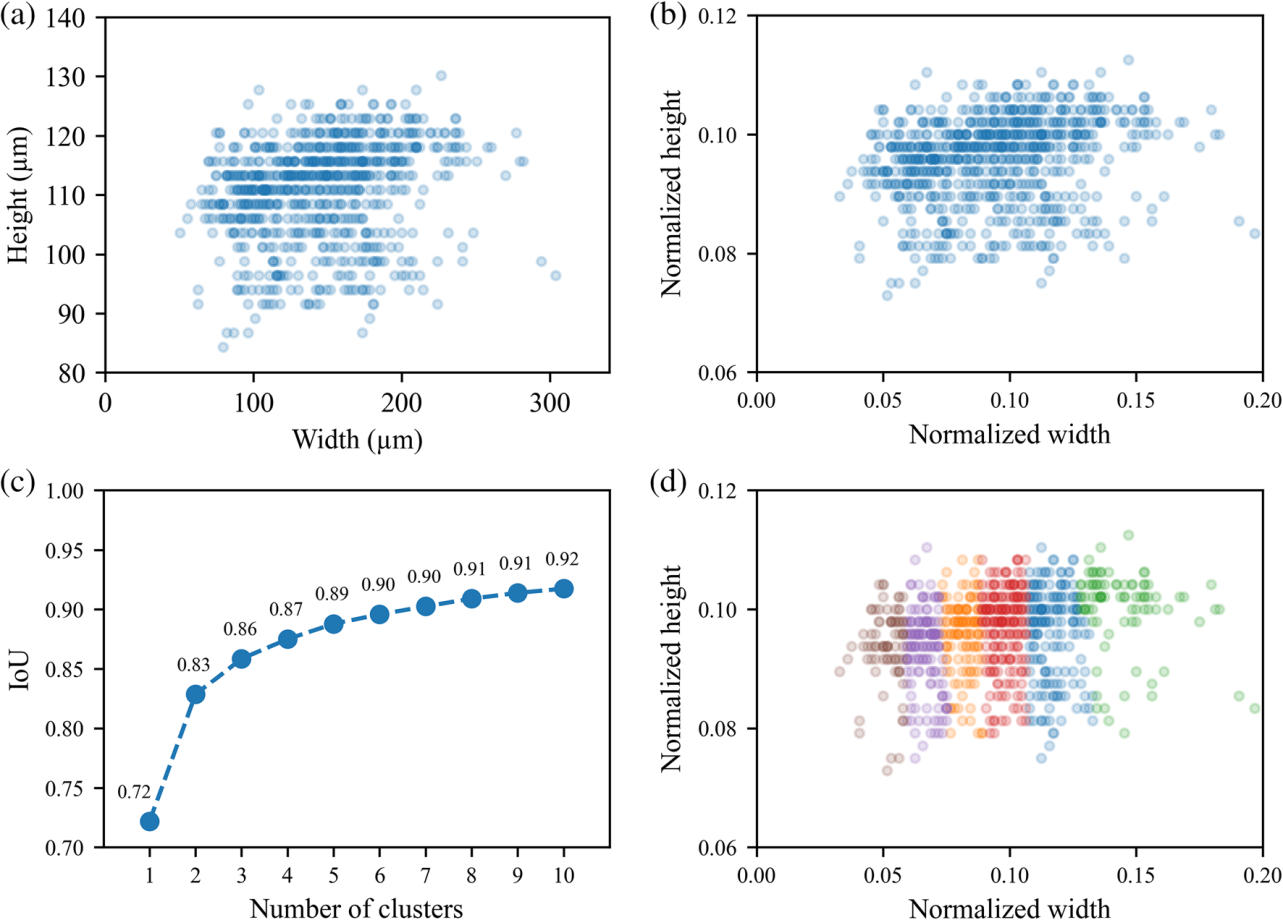
K-means clustering for YOLOv3 anchor boxes: widths and heights of ground truth bounding boxes (**a**) in µm and (**b**) normalized to the image size; (**c**) IoU for different numbers of clusters; (**d**) k-means clustered data set into six clusters.

To maximize the accuracy of proposing bounding boxes, the data set ground through bounding boxes were analyzed using the standard unsupervised learning technique k-means clustering, with intersection over union (IoU) metrics. A total of 814 ground truth bounding boxes were analyzed (Fig. 9a), and their widths and heights were normalized based on the image resolution (Fig. 9b). Then, k-means clustering was performed, and the IoU was calculated between corresponding centroids and data points for each cluster. As the number of clusters increases (Fig. 9c), the IoU also increases, showing a plateau effect, which means the number of clusters should be chosen wisely. Higher IoU is better for accuracy; however, when more anchor boxes are used, the number of convolutions in detection layers increases along with the YOLO model size and computational cost. For future training, six clusters with bounding anchor boxes were chosen, as shown in Table 4. The clustered data set into six clusters is shown in Fig. 9d.

**Table 4:** Anchor box sizes chosen by k-means clustering.

Cluster no	Normalized centroid for each cluster (w × h)	416 × 416 pixel resolution anchor box size (w × h), pixels	640 × 480 pixel resolution anchor box size (w × h), pixels
1	0.0513 × 0.0937	21 × 39	33 × 45
2	0.0665 × 0.0930	28 × 39	43 × 45
3	0.0816 × 0.0956	34 × 40	52 × 46
4	0.0979 × 0.0969	41 × 40	63 × 47
5	0.1167 × 0.0960	49 × 40	75 × 46
6	0.1448 × 0.0999	60 × 42	93 × 48

### Training and validation

**Figure 10:**
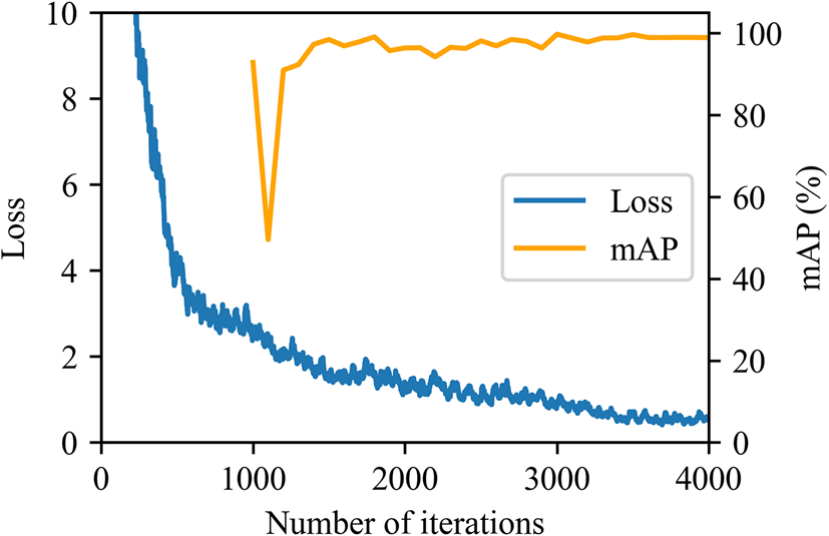
YOLOv3 object detection model training process.

**Figure 11:**
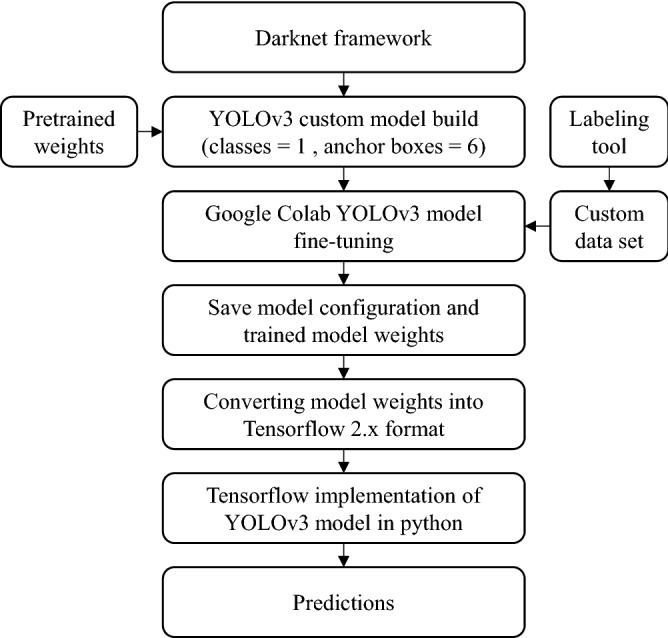
Flowchart of the YOLOv3 object detection model fine-tuning and predictions.

The training was performed using the Darknet framework to build the YOLOv3 model structure with customized layers. First, the customized model was constructed, and model weights pretrained on the COCO image data set were loaded. Second, training was conducted for 4,000 iterations using the training data set, as described in Sect. 2. While the loss was converging to the minimum, the mean average precision (mAP) was calculated every 100 epochs starting from 1000 epoch showing the model’s ability to perform on validation data set, which is illustrated in Fig. 10. Third and last, the best weights with the highest mAP during the training were saved, and the TensorFlow implementation model was built for predictions using the YOLOv3 configuration file. Figure 11 shows the flowchart of this process.

### Printing results defect detection

**Figure 12:**
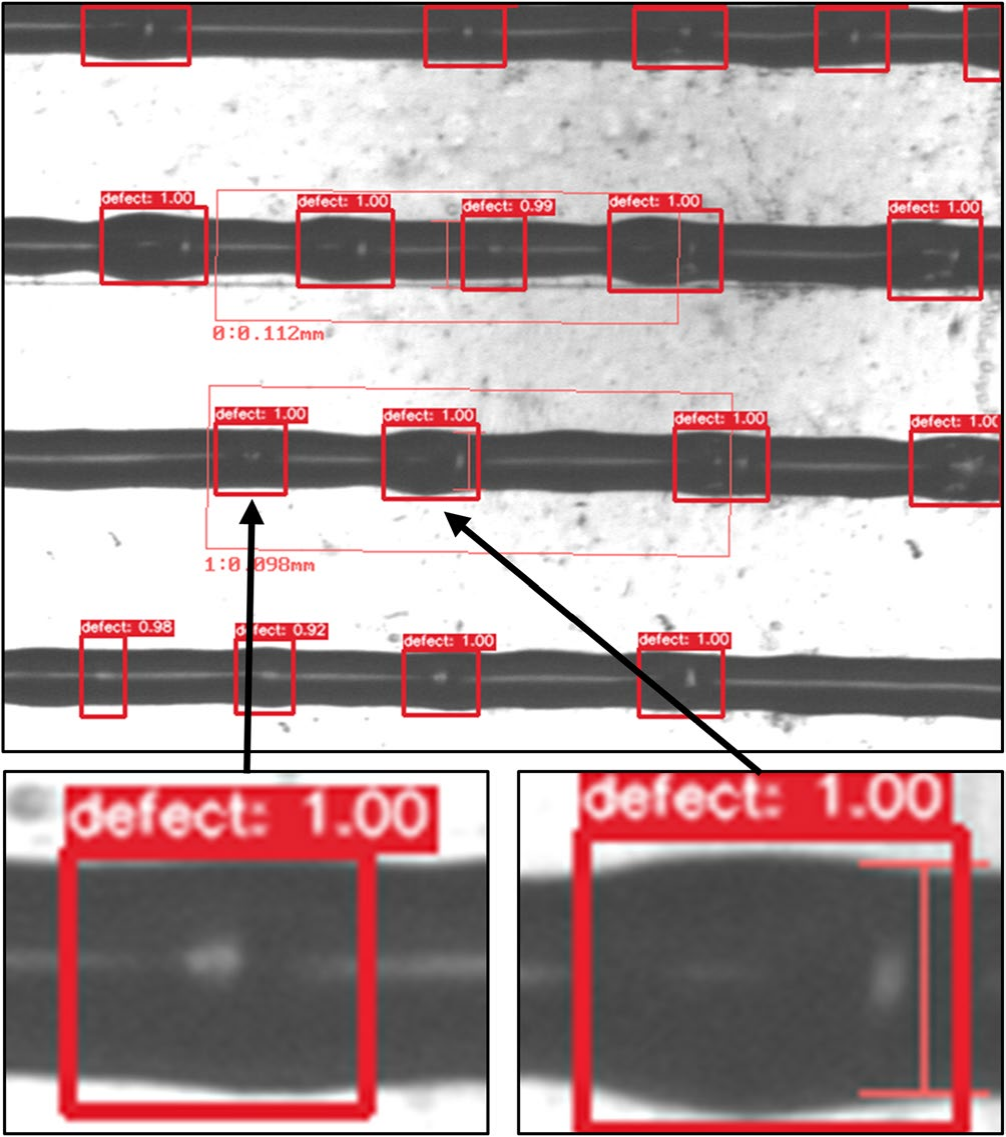
Detection example of YOLOv3 retrained object detection model.

**Figure 13:**
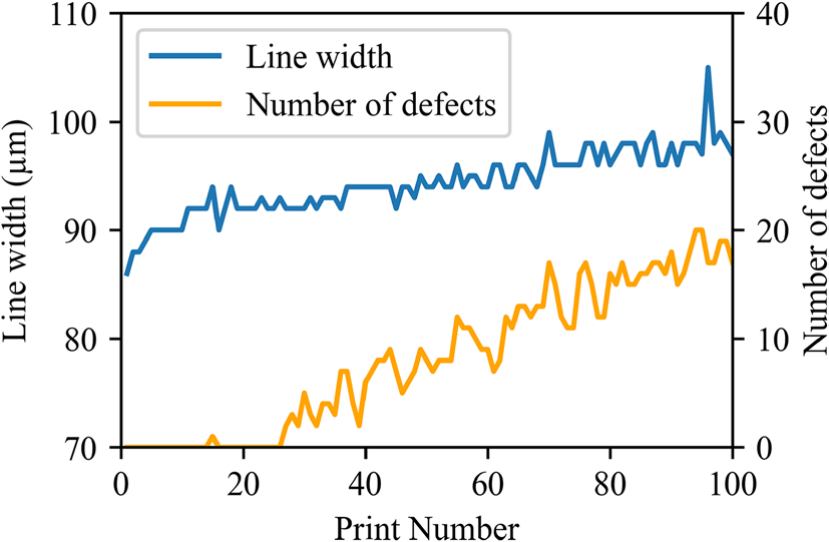
The measured line width of printed lines augmented by the number of local defects.

As a result, the printing defect detector could predict printing defects within the captured image, as shown in Fig. 12. It is possible to decide whether the observed image of the printed pattern is of satisfactory quality or not after obtaining information about defects. In particular, the most straightforward way of doing this is to count the number of defects regardless of their position or size. Figure 13 shows the printing results based on line width measuring, which were augmented with the number of local defects detected by the AI model. It is seen that as the line width increases, the defects start to appear. Thus, the failure regime may be detected more reliably. The inference time of the model is critical when integrated with higher yield production lines. When the model was run by the PC (Core i7-6700 3.4 GHz processor and NVidia GeForce GTX 750 Ti 1 Gb RAM graphics card), 356 ± 18 ms were taken for inference of each image.

## Conclusion

During printed electronics production, printing consistency is of crucial importance. The instability of the materials’ properties affects printing process reliability and causes printing defects. Aside from preventive measures, tools for monitoring the actual state should be in place, and the most common method is measuring line width increases that may indicate unacceptable printing quality. However, while measured line width gain is acceptable, other defects may be obscured from the conventional computer vision system unless its complexity significantly increases. This issue can be addressed to the vision system assisted by AI computer vision tools, namely, overall printing quality and defect detection based on AI DNNs, as promulgated in this paper. It took 356 ± 18 ms to infer each image. We hope that the proposed method will help for printing reliability assessment through detecting defective prints and later can be merged with the printing control algorithms or be used as an archiving tool for quality certification purposes.

## Data Availability

Image data set for this study can be found at: https://data.mendeley.com/datasets/fpf2jv378d/draft?a=8ef6e4c8-c3f4-40c5-b948-e73db1f0e7c2.
